# Judgement Safeguarding the Interests of Children and Pregnant Women Convicts in Indian Jails

**DOI:** 10.4103/0970-0218.40889

**Published:** 2008-04

**Authors:** RK Bansal, Anupam Verma, Manoj Bansal

**Affiliations:** Surat Municipal Institute of Medical Education and Research, Surat - 395 010; U.N. Mehta Institute of Cardiology and Research Centre, Ahmedabad - 380 016, Gujarat, India. E-mail: drrkbansal@gmail.com

Sir,

Recently,(1) the Supreme Court of India took cognizance of the plight of children living in jails because of the arrest of their mothers and delivered a landmark judgement greatly strengthening children's and pregnant women's rights. The Chief Justice, Mr. Y. K. Sabharwal, was moved by the plight of such children, who for none of their fault, but per force, have to stay in jail with their mothers.

The court took notice of the Constitutional provisions of India, Articles 21, 23, 39 (e), 39 (f), 21 A, 14, 42, 45, 47; existing laws concerning children; international laws and conventions; scientific reports of institutions and committees; affidavits of State governments and Union Territories. It was brought out that there were 6496 under-trial women with 1053 children and 1873 convicted women with 206 children. The Court noted situations of neglect and issued guidelines for adequate protection of children, pregnant women and childbirth in prison. The court declared food, clothing, medical care, shelter, education and recreation as child's rights and issued detailed guidelines in upholding the health of children and women in jails. The court directed that jail manual and relevant rules be amended within 3 months to comply with its directives and periodical inspections to ascertain compliance with its judgements in letter and spirit. The court directed the Union of India, State governments, Union territories and State legal services authorities to submit a compliance report stating the steps taken within 4 months. The court declared that the Constitution casts an obligation to the State to look after the welfare of children and provide for social, educational and cultural development of the child with its dignity intact and protected from any kind of exploitation.

The court also directed priority disposal of criminal trials of women prisoners whose children are in jail. The court stated that international laws and conventions are enforceable when they elucidate and effectuate fundamental rights, and can be read as part of domestic law if mutually consistent.

The judgement is indeed laudable with hopes of a better future for such children. In the modern era, there is no doubt that every child throughout the world is entitled to a healthy environment full of freedom and dignity. As our Supreme Court succinctly puts it, “every citizen is entitled to the finer graces of civilization” under Article 21 of the Indian Constitution(2) and it would be delightful to witness this concept extending to children in prison.
